# Cystatin C-Based Evaluation of Kidney Function of HIV-Infected Children in Benin City, Southern Nigeria

**DOI:** 10.1155/2012/861296

**Published:** 2012-11-19

**Authors:** Moses Temidayo Abiodun, Nosakhare J. Iduoriyekemwen, Phillip O. Abiodun

**Affiliations:** Department of Child Health, University of Benin Teaching Hospital, Benin 300001, Nigeria

## Abstract

*Background*. Human immunodeficiency virus (HIV) is now a confirmed risk factor for kidney disease with an increased burden in persons of African descent. *Method*. We measured the serum cystatin C levels of 205 ART-naive, HIV-infected children by an ELISA technique and compared them with the levels of apparently healthy children. *Result*. The mean ± SD serum cystatin C level of children with HIV infection was 1.01 ± 0.44 mg/L, significantly higher than the mean value in the control group, that is, 0.72 ± 0.20 mg/L (*P* = 0.000). The mean ± SD cystatin C-based estimated GFR of children with HIV infection was 102.7 ± 31.0 mL/min/1.73 m^2^, significantly lower than 126.9 ± 28.5 mL/min/1.73 m^2^ in the control group, (*P* = 0.014). A significantly higher proportion of HIV-infected children compared to controls had eGFR < 90 mL/min/1.73 m^2^ (21.5% versus 5.4%; *P* = 0.00). The prevalence of chronic kidney disease (CKD) among the HIV-infected children was 10.7%. The cystatin C-based eGFR of the HIV-infected children ≥5 years old correlated positively with their CD4 count (*r* = 0.23;  *P* = 0.022). *Conclusion*. There is a high prevalence of CKD among HIV-infected children, requiring regular monitoring of their kidney function using a cystatin C-based method.

## 1. Introduction

HIV-related kidney diseases have been reported in children since 1989 by Strauss and his colleagues [1]. Chronic kidney disease is now a major cause of morbidity among HIV-infected children since comprehensive HIV care with highly active antiretroviral therapy (HAART) and prompt treatment of opportunistic infections enable patients to live long enough to develop kidney damage. Early stages of HIV-related chronic kidney disease are usually asymptomatic, often manifesting with mild reduction in glomerular filtration rate (GFR). It is therefore essential to make early diagnosis and institute treatment that will prevent further decline to end stage renal disease (ESRD) requiring renal replacement therapy. This is more pertinent in resource-poor African settings where facilities for renal replacement therapy are limited and very expensive [2, 3].

Serum creatinine which is commonly used in the estimation of GFR is inaccurate due to the effects of diet, gender, race, tubular secretion, and increased extrarenal elimination found often in advanced HIV disease [4, 5]. Also, muscle wasting is prevalent in HIV-infected African children, further diminishing the validity of creatinine as a measure of kidney function in this cohort [6, 7]. Conversely, cystatin C, a 120-amino acid, 13-kDa protein belonging to the cystatin super-family of competitive inhibitors of lysosomal cysteine proteases, has a constant production rate by all nucleated cells, is freely filtered at the glomeruli, and has no systemic reabsorption or renal tubular secretion [8–10]. In addition, its serum level is not significantly influenced by age, gender, or muscle mass. Hence, cystatin C is superior to creatinine as a marker of kidney functions. Likewise, cystatin C-based formulae would mirror gold-standard measures of GFR and reflect impaired kidney function earlier than creatinine-based equations [7, 11, 12].

 In view of the foregoing, this study evaluated the kidney function of HIV-infected children using a serum cystatin C-based method so that early stages of CKD could be accurately and promptly detected to forestall ESRD.

## 2. Methods

### 2.1. Study Setting and Participants

 The study was carried out from May to October 2011 at the paediatric outpatient (POP) HIV clinics of the only three public hospitals (University of Benin Teaching Hospital, Central Hospital, and Stella Obasanjo Children Hospital) providing medical care including antiretroviral therapy (ART) for children with HIV infection in Benin City, Southern Nigeria. The clinics are supported by the US President's Emergency Plan for AIDs Relief (PEPFAR) and non-governmental organizations. It was a cross-sectional study, approved by the Ethical Committee of the University of Benin Teaching Hospital. Written informed consent was obtained from parents/guardians and assent from children ten years old and above.

The HIV status of the participants was determined according to the WHO standardized serial testing algorithm using Determine HIV-1/2 (Abbott, Tokyo, Japan), followed by Uni-Gold Recombigen HIV (Trinity Biotech, Wicklow, Ireland) for a positive test. HIV-1/2 STAT-PAK (Chembio, Medford, NY) was used as a “tie-breaker” for sero-discordant results [13]. Diagnosis was based on documented positive HIV DNA PCR tests on two separate samples, for those who were diagnosed before the age of 18 months. All consecutive ART-naïve HIV-infected children, aged 18 months to 17 years at the POP clinics, were recruited. Altogether, 227 HIV-infected children attended the POP's clinics during the study period; 22 were excluded: nine required immediate admission for acute illnesses; seven samples were discarded due to haemolysis; six had no CD4+ cell count/percent results because of insufficient or spilled samples. Thus, 205 HIV-infected children were enrolled. 

Also, 205 age- and sex-matched apparently healthy HIV-negative children who were on follow up at General out-patient clinics of the 3 public hospitals were enrolled as controls. Age matching was done to the nearest half year for those <5 years and to the nearest one year for those ≥5 years. The matching was done prior to analysis of the samples for serum cystatin C to avoid bias. Patients who had sickle cell disease, cardiac disease, previously diagnosed kidney disease, and those on systemic steroid were excluded.

### 2.2. Data Collection and Clinical Evaluation

Participants were recruited consecutively from the 3 public hospitals in a ratio of 5 : 2 : 1, respectively, based on their varied patient loads. Relevant sociodemographic and clinical information were obtained. The socioeconomic classification of the participants was based on maternal education and paternal occupation [14]. They were thoroughly examined. Also, their chest radiographs were reviewed to detect pulmonary tuberculosis. Children with HIV infection were staged according to the revised WHO paediatric clinical staging criteria [15]. In this study, subjects were further described as having “advanced” or “not advanced” HIV disease [16]. Advanced HIV disease was defined as WHO clinical stages 3 or 4, CD4 percent < 25% (in children < 5 years), or CD4+ T cell count <350 cells/mm^3^ (in children ≥ 5 years).

### 2.3. Sampling and Laboratory Procedure

Five milliliters (5 mL) of blood was obtained from every study participant. Two milliliters out of the 5 mL of blood was allowed to clot, while the resulting serum was frozen at −8°C after centrifugation until analysis for serum cystatin C. The remaining 3 mL of blood was analysed in the PEPFAR/UBTH laboratory for CD4+ cell count or percent in the HIV-infected children, detection of HIV antibodies in the controls, and haemoglobin electrophoresis in both groups. All samples were transported from the sites of collection to the PEPFAR/UBTH laboratory in an ice-packed polythene container with cock-screwed lid within an hour of collection. Specimen containers were coded to ensure confidentiality.

In batches of 82 samples, cystatin C was measured quantitatively by a Sandwich enzyme-linked immunosorbent assay (ELISA) technique, using kits manufactured by WKEA Med Supplies Corps, Changchun China with a sensitivity of 0.01 *μ*g/L, specificity 100%, assay range: 30 *μ*g/L–800 *μ*g/L and LOT 20110726. The end product was measured spectrophotometrically at 450 nm using a micro-well Diareader ELX800UV (Dialab GmbH Vienna/Austria). The actual concentration of serum cystatin C in the sample was obtained by multiplying the measured cystatin C concentration by a dilution factor of 5 [17]. Serum cystatin C level greater than 1.00 mg/L was classified as elevated as in previous studies [7, 16]. The GFR (mL/min/1.73 m^2^) was estimated by the cystatin C-based equation derived by Filler and Lepage [11]: Log (GFR) = 1.962 + [1.123 ∗ log (1/cystatin C)], where 1/cystatin C is the reciprocal of the concentration of serum cystatin C in mg/L.

### 2.4. Statistical Analysis

 The data were analysed using the Software Package for Social Science (SPSS) version 17.0 (Windows Inc; Chicago, IL, USA). Anthropometric *z*-scores were calculated using the WHO AnthroPlus software developed using the WHO Child Growth Standards and the WHO Reference 2009 [18]. Then, the *z*-scores of the HIV-positive group were compared to those of the controls using the Mann-Whitney *U*-test. 

 Continuous data such as age, cystatin C, and eGFR were summarized as mean (±SD), while categorized data like sex, socioeconomic class, and HIV stages were represented as proportions. Fisher's exact test or Pearson's chi-square was used to compare categorized data while Student *t* test was used to assess for any significant difference between two means. A 2-sided *P* value < 0.05 was considered significant. Pearson's correlation test was done for the association between eGFR and CD4+ cell count.

## 3. Results

### 3.1. Demographic and Clinical Characteristics of Study Participants

A total of 205 children infected with HIV (98 males and 107 females) and 205 uninfected ones were studied. The mean ± SD age of the children infected with HIV was 5.9 ± 3.5 years while that of the control group was 5.4 ± 3.5 years. The youngest study participant was 1.5 years while the oldest was 17 years. Among the 205 HIV-infected children 11 (5.4%) were in upper socioeconomic class (SEC), 83 (40.5%) in middle class, and 111 (54.1%) in lower class, with a significantly larger proportion in the lower SEC compared to the controls, (*χ* ^2^ = 10.77, *df* = 2, *P* = 0.005). The HIV-infected children were leaner (BMI *z*-score −0.83 versus 0.40; *P* = 0.002), shorter (HAZ score −0.65 versus 0.02; *P* = 0.000) and lighter (WAZ score −1.015 versus −0.02; *P* = 0.000) compared with the control (Table 1).

Based on WHO paediatric clinical staging system, 129 children (62.9%) had stage 1 or 2 disease (not advanced) while 76 (37.1%) had stage 3 or 4 disease (advanced). Using WHO immunological staging, 41 (38.7%) of the HIV-infected children younger than 5 years had CD4+ cell percent <25% while 23 (23.2%) of children ≥5 years had a CD4+ cell count less than 350 cells/mm^3^, both classified as advanced disease.

### 3.2. Serum Cystatin C Levels of All Study Participants

The mean serum cystatin C level was 1.01 ± 0.44 mg/L in the HIV-infected group compared with 0.72 ± 0.20 mg/L in the control group (*P*-value = 0.000). Similarly, 48 (23.4%) HIV infected children compared with 11 (5.4%) in the control group had serum cystatin C > 1 mg/L (*P*-value = 0.000). In all age groups, the mean serum cystatin levels were significantly higher in the HIV-infected children than in the controls (*P* < 0.05, Table 2).

The correlation of age, weight, height, and BMI with serum cystatin C was not significant in both groups of children. Also, there was no significant correlation of CD4% with cystatin C in children less than 5 year old (*r* = −0.184; *P* = 0.059). However, CD4+ cell count correlated significantly with cystatin C in the HIV-infected children older than 5 years (*r* = −0.281; *P* = 0.005). 

### 3.3. Cystatin C-Based Estimated Glomerular Filtration Rates of Study Participants

The mean estimated glomerular filtration rate (eGFR) of HIV infected children was 102.7 ± 31.0 mL/min/1.73 m^2^ which was significantly lower than the value of 126.9 ± 28.5 mL/min/1.73 m^2^ in the control group (*P* = 0.014). Also, 44 (21.5%) children with HIV infection had reduced eGFR < 90 mL/min/173 m^2^ compared to 11 (5.4%) in the control group. Altogether, significantly higher proportions of the HIV-infected children than the controls had eGFR < 90 mL/min/173 m^2^ (*P* = 0.000; Table 3).

The prevalence of chronic kidney disease (CKD) among HIV-infected children in this study was 10.7%. Children infected with HIV were 12.2 times more likely to have CKD compared to the controls (Fisher's exact test = 17.703; OR = 12.202; 95% C.I. = 2.830–52.608; significant at *P* < 0.05).

Children with HIV infection who had CKD were similar in mean age and body mass indices to those without CKD. The peak age for CKD was 5–9 years. Those with CKD had a male-female rate of 1.2 : 1, and 13 (59.0%) of them were in advanced immunological stage of HIV disease. Also, they had a significantly lower mean CD4 count and higher serum cystatin C compared to others. CD4 count < 200 was predictive of CKD in HIV-infected children (OR = 5.926; C.I. =1.403–25.028; *P* = 0.025; Table 4).

### 3.4. Relationship between Kidney Function and Paediatric Aids Stages of the HIV-Infected Children

There was a statistically significant association between estimated glomerular filtration rates (eGFRs) and immunological stages of HIV-infected children ≥ 5 year old (*P* = 0.028). However, there was no such association in those <5 years (*P* = 0.112; Table 5). Also, there was no significant association between eGFR and clinical stages of HIV infection at all ages: (<5 years old: *df* = 3; *χ* = 2.801, *P* = 0.462; ≥5 years: *df* = 3,  *χ* = 4.897, *P* = 0.153).

**Figure 1:**
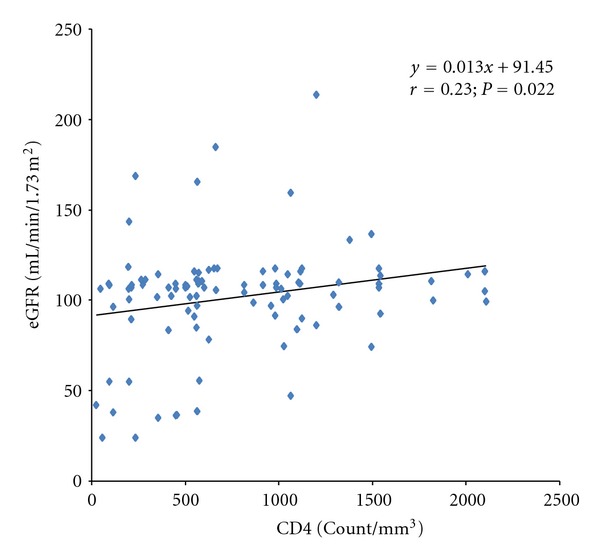
Correlation of estimated glomerular filtration rate with CD4 count in HIV-infected children ≥ 5 years old.

There was a significant positive correlation of kidney function (assessed using cystatin C-based eGFR) with CD4 count in children who were 5 years and older in this study (*r* = 0.23; *P* = 0.022; Figure 1).

## 4. Discussion

This study confirmed high levels of serum cystatin C in HIV-infected children. Their mean serum cystatin C (1.01 ± 0.44 mg/L) is higher than the mean value (0.77 ± 0.29 mg/L) obtained by Esezobor et al. [16] in HIV-infected children in Lagos. The difference could be partly due to the larger proportion of children with elevated serum cystatin C (>1 mg/L) in this study than in the Lagos study (23.4% versus 16.7%). However, the high level of serum cystatin C of HIV-infected children found in this study is similar to the mean value (1.03 ± 0.02 mg/L) found by Jones et al. [7] among 250 HIV-positive adults in the Nutrition for Healthy Living (NFHL) study. Also, it is consistent with the values (0.93 + 0.32 mg/L and 0.92 + 0.22 mg/L) obtained by Jaroszewicz et al. [19] in Poland and Odden et al. [20] in the USA, respectively. Furthermore, the high mean serum cystatin C in this work is consistent with prior reports of increased burden of HIV-related kidney diseases in blacks [21].

Compared to the controls, the HIV-infected children had significantly higher serum cystatin C levels in all age groups in this study. This is consistent with the findings of Esezobor et al. [16] and reports from other regions of the world [19, 20]. This trend suggests that the kidneys, while serving as reservoirs for HIV [22], suffer subclinical damage causing persistently elevated serum cystatin C in HIV-infected persons. Thus, nearly a quarter of the children with HIV infection in this survey had elevated serum cystatin C (>1 mg/L) which is prognostic of poor renal and cardiovascular outcomes as well as death in this cohort [23]. It is important to note that the serum cystatin C levels of the controls in all age groups consistently remain within the reference ranges published by Finney et al. [10] for healthy children. Since cystatin C has no significant extraglomerular excretion routes, the stable level obtained in these controls reflects the constancy of GFR in healthy children after infancy.

This study found no correlation between cystatin C and body mass indices of the participants, confirming previous reports in an African study [16] and researches from other regions [11, 24]. This implies that cystatin C remained an accurate marker of kidney function in the general population and even among HIV-infected African children who are often malnourished. In this study, only CD4 count significantly correlated with serum cystatin C. This is contrary to the findings of Jaroszewicz et al. [19] in Polish adults who found no correlation between serum cystatin C levels and CD4 counts. This may be due to the dual effects of ART on the serum cystatin C levels and CD4 counts of their study participants [19].

The mean cystatin C-based eGFR of the HIV positive group was 102.7 ± 31.0 mL/min/1.73 m^2^, significantly lower than that of the age and sex matched controls consistent with previous reports [16, 25]. Also, the fact that 21.5% of the HIV-infected group had cystatin C-based eGFR below normal range (<90 mL/min/1.73 m^2^) further attests to the detrimental effects of HIV on kidney function. Hence, the HIV Medicine Association of the infectious Disease Society of America (IDSA) recommended regular monitoring of GFR in HIV-infected children in order to promptly detect renal impairment and institute measures that may halt or slow its progression [24]. Also, an accurate measure of GFR with serum cystatin C will enable recommended drug dosage adjustments to avoid systemic toxicities of drugs excreted via the kidneys [26]. Therefore, as shown in this work, accurate measure of GFR in HIV infection before therapy and during follow-up is pertinent to a good renal outcome.

The high prevalence of CKD (10.7%) among HIV-infected children in this work is consistent with the report of the previous Nigerian study of HIV-infected children using a cystatin C-based method [16]. This is higher than the prevalence of 5.1% obtained by Iduoriyekenwen et al. [27] recently in HIV-infected children in one of the study centres (UBTH) using their creatinine-based eGFRs. This further confirms the superiority of cystatin C to creatinine in this cohort. However, the prevalence in the present study is lower than the 15.2% found by Jones et al. [7] among 250 HIV-infected adults using cystatin C-based eGFR. The higher prevalence of CKD observed by Jones et al. [7] was apparently due to longer duration of HIV disease, use of nephrotoxic drugs, and comorbidities like liver disease, diabetes mellitus and viral hepatitis in some of their study participants [7]. Cystatin C levels can also be influenced by nonrenal factors including systemic inflammation [7].

HIV infected children are more likely to develop CKD as observed in this study (OR = 12.202; C.I. = 2.830–52.608; *P* < 0.05), consistent with previous reports of increased burden of CKD among HIV-infected children highlighting an increasing need for renal replacement therapy which the health system is currently ill-prepared for in this sub-region [2, 3]. There was male preponderance among children with CKD in the study, consistent with prior reports among Africans [28, 29]. The peak age group (5–9 years) for CKD in this study coincided with that reported by Anochie et al. [29] among children with HIV-related CKD in Port Harcourt. This may be due to the fact that a majority of perinatally infected children followed a downhill course with complications involving the kidneys and other organs by school age, or perhaps due to similar virulence/nephropathic effect of HIV serotypes prevalent in this region. Moreover, consistent with prior reports [16, 21], children with HIV infection in this study were significantly leaner than the controls, and more likely to be in the lower social economic class, portraying poverty-malnutrition-disease cycle. 

Other significant predictors of CKD found in this study include nadir CD4 count < 200 cells/mm^3^, consistent with the findings of Emem et al. [30] in a multicentre study in southern Nigerian HIV-infected persons. Also, Jones et al. [7] and other researchers [18, 21] in the USA found that low CD4 count and/or elevated serum cystatin C were predictive of kidney disease and sometimes death in HIV infected persons. Furthermore, Jaroszewicz et al. [19] in Poland identified high viral loads and prolonged use of ART as risk factors for CKD. However, the viral loads of the children recruited in this study were not determined.

The eGFR of the HIV-infected children positively correlated with their CD4 count in this study, contrary to the report of the previous Nigerian study which found no relationship between the kidney function and immunological/clinical stages of HIV-infected children, possibly underpowered by a small sample size. Emem et al. [30] and other investigators [7, 29, 31] confirmed worsening of kidney function in advanced immunological stages of HIV disease. Surprisingly, the correlation of kidney function with immune status was not significant in children less than 5 years old, possibly due to the shorter duration of their HIV infection [31].

In this study, kidney function showed no significant association with clinical stages of HIV disease in all age groups, despite its correlation with the immunological stages of the children older than 5 years. This is an instance of clinical-immunological discordance in HIV infection consistent with the report of  Wools-Kaloustian et al. [32] that renal impairment was significantly associated with severe immunosuppression, but independent of several parameters for clinical staging of HIV infection. This may be due to the fact that common comorbidities like tuberculosis, found in over 10% of the seropositive group in this study, classify patients into advanced clinical stages, irrespective of their renal or immunological status. Conversely, renal impairment may be the first manifestation of HIV disease in some patients. Winston et al. [31] found in a large series that about 50% of patients later confirmed to have severe immunosuppression and HIVAN were otherwise clinically asymptomatic at presentation. Thus, they were inadvertently classified into early clinical stages of HIV disease until further evaluation confirmed advanced immunological stages and renal disease [31]. The strengths of our study are the recruitment of only ART-naïve patients and the analysis of serum cystatin C in a single laboratory, avoiding systematic errors. 

The limitations of this study include the lack of assessment of the cause of CKD and the lack of data on proteinuria, an important marker of kidney damage [33]. Also, we used a single measurement of GFRs without repeating 3 or more months later to exclude transient reduction in GFR [33]. Cystatin C was measured with a nephelometric assay to derive the Filler's formula [11]. However, we measured cystatin C with an enzyme-linked immunosorbent assay. Also, like other formulae for the estimation of GFR, the Filler's formula has not been validated in HIV-infected children. 

In conclusion, there was a high prevalence of CKD among the HIV-infected children and nadir CD4 count < 200 cells/mm^3^ was a potent risk factor for CKD in this study. Children aged ≥ 5 years with advanced immunological stages of HIV disease had significantly worse kidney function than those in earlier stages. The high burden of CKD and elevated serum cystatin C indicative of early renal dysfunction found in this study should be taken into cognizance while providing basic health care/ART programmes in this medically under-served region of the world.

## Figures and Tables

**Table 1:** Demographic and clinical characteristics of all study participants.

Characteristics	HIV-infected children, *n* = 205 (%)	Controls, *n* = 205 (%)	Test (*χ* ^2^, *t*, *U*)	*df*	*P* value
Age (years)					
Less than 5 years	106 (51.7)	107 (52.2)	0.0098	1	1.000^a^
≥5 years	99 (48.3)	98 (47.8)			
Mean age	5.68 ± 3.41	5.39 ± 3.41	0.84	408	0.401^b^
Sex					
Male	98 (47.8)	98 (47.8)	0.00	1	1.000^a^
Female	107 (52.2)	107 (52.2)			
SEC					
Upper class	11 (5.40)	31 (14.6)	10.77	2	0.005^c∗^
Middle class	83 (40.50)	78 (38.0)			
Lower class	111 (54.10)	96 (47.30)			
Weight					
Mean ± SD (kg)	18.3 ± 7.7	19.5 ± 9.0	−1.327	408	0.171^b^
WAZ	−1.015	−0.20	13043.5		0.000^d∗^
Height					
Mean ± SD (m)	1.093 ± 0.197	1.093 ± 0.218	0.012	408	0.991^b^
HAZ	−0.65	0.02	15159.5		0.000^d∗^
Body mass index					
Mean ± SD (kg/m^2^)	14.91 ± 3.54	15.68 ± 3.34	2.265	408	0.024^b∗^
BMI *z *	−0.83	0.40	17221.0		0.002^d∗^
Blood pressure					
Mean ± SD (mmHg)	86.4 ± 2.1	86.2 ± 3.2	0.7482	408	0.4548^b^

WAZ: weight for age *z* score; HAZ: height for age *z* score; BMI *z*: body mass index *z* score; SEC: socioeconomic class; ^a^Fishers exact test; ^b^student *t* test;^ c^Pearson chi-square; ^d^Mann-Whitney *U* test; *significant at *P* < 0.05.

**Table 2:** Serum cystatin C levels of all study participants in different age groups.

	Serum cystatin C (mg/L)						
Age groups(years)	HIV-infected		Controls		Test (*t*)	*df*	*P* value
	*n*	Mean ± SD	*n*	Mean ± SD			
1–4	106	1.00 ± 0.46	107	0.74 ± 0.20	5.49	211	0.000*
5–9	72	1.03 ± 0.43	74	0.69 ± 0.18	6.134	144	0.000*
10–14	23	1.01 ± 0.44	20	0.77 ± 0.28	2.103	41	0.042*
15–17	4	0.89 ± 0.09	4	0.65 ± 0.17	2.481	6	0.048*

*t*: student's *t* test, *df*: degree of freedom, *significant at *P* < 0.05.

**Table 3:** Cystatin C-based estimated glomerular filtration rates of all study participants.

eGFR	HIV-infected children, *n* = 205 (%)	Control *n* = 205 (%)	*P* value
Mean ± SD	102.7 ± 31.0	126.9 ± 28.5	0.014^a∗^
Category			
≥90	161 (78.5)	194 (94.6%)	
89–60	22 (10.7)	9 (4.4%)	0.000^b∗^
59–25	19 (9.3)	2 (1.0%)	
15–24	3 (1.5)	0 (0.0)	

eGFR: estimated glomerular filtration rate (mL/min/1.73 m^2^); ^a^student *t* test; ^b^Fisher's exact = 26.045, *df* = 3; *significant at *P* < 0.05.

**Table 4:** Comparison of characteristics between HIV-infected children with and without chronic kidney disease.

Characteristics	HIV-infected children		Test (*t*, *χ*2, *U*)	*df*	*P* value
	With CKD *n* = 22, (%)	Without CKD *n* = 183, (%)			
Age					
Mean ± SD	5.77 ± 3.463	5.66 ± 3.407	0.141	203	0.888^a^
BMI *z* score	−0.155	−0.42	1904.0		0.678^b^
Immunological stage					
Advanced	13 (59.1)	49 (26.8)	9.721	1	0.0031^c∗^
Not advanced	9 (40.9)	134 (73.2)			
CD4 count					
Mean ± SD (cells/mm^3^)	348.8 ± 297.5	844.8 ± 532.0	−3.153	97	0.002^a∗^
Cystatin C					
Mean ± SD (mg/L)	2.15 ± 0.48	0.87 ± 0.13	28.04	203	0.000^a∗^

CKD: chronic kidney disease. ^a^Student's *t*-test;^ b^Mann-Whitney *U* test; ^c^Fisher's exact test; *significant at *P* < 0.05.

**Table 5:** Estimated GFR of HIV-infected children in different immunological stages.

eGFR category	Immunological stages		Total	*P* value
	Not advanced	Advanced		
<5 year old				
≥90	52 (80.0)	29 (70.7)	81 (76.4)	0.112^a1^
60–89	10 (15.4)	5 (12.2)	15 (13.2)	
25–59	3 (4.6)	4 (9.8)	7 (7.5)	
15–24	0 (0.0)	3 (7.3)	3 (2.8)	

Total	65 (100.0)	41 (100.0)	106 (100.0)	

≥5 year old				
≥90	63 (82.9)	17 (84.6)	80 (80.8)	
60–89	7 (9.2)	0 (0.0)	7 (7.1)	0.028^a2∗^
25–59	6 (7.9)	6 (15.4)	12 (12.1)	

Total	76 (100.0)	23 (100.0)	99 (100.0)	

eGFR: estimated glomerular filtration rate (mL/min/1.73 m^2^); ^a1^Fisher's exact = 5.693; *df* = 3; ^a2^Fisher's exact = 6.192; *df* = 2; *significant at *P* < 0.05.
